# Unveiling the Exodus: A scoping review of attrition in allied health

**DOI:** 10.1371/journal.pone.0308302

**Published:** 2024-09-06

**Authors:** Su Ann Yeoh, Saravana Kumar, Anna Phillips, Lok Sze Katrina Li

**Affiliations:** 1 UniSA Allied Health & Human Performance, University of South Australia, Adelaide, Australia; 2 School of Allied Health, Human Services and Sport, La Trobe University, Melbourne, Australia; Endeavour College of Natural Health, AUSTRALIA

## Abstract

**Background:**

Efficient utilisation of allied health workforce may help address the predicted shortfall of 18 million health workers estimated by 2030. Knowledge about allied health professionals’ attrition, or intention to leave, and factors influencing attrition can assist in developing evidence-informed strategies to mitigate this issue. The review aimed to map attrition and attrition intention rates, and its attributing factors for allied health professions worldwide.

**Methods:**

Adhering to the PRISMA-ScR guidelines, a comprehensive search was conducted across academic databases (PsycINFO, MEDLINE, Embase, Emcare, CINAHL, Scopus, and the Cochrane Library database) and grey literature (Google, Google Scholar, organisational websites). Two reviewers independently undertook a two-stage screening process along with data extraction using customised data extraction forms. A narrative synthesis was used to synthesise the data.

**Results:**

Thirty-two studies published between 1990 and 2024 were included. Attrition rates ranged from 0.5% to 41% across allied health disciplines. Pharmacists demonstrated the lowest attrition rates, while audiologists reported the highest. Radiographers reported the lowest intent to leave at 7.6%, while occupational therapists showed highest intent to leave, ranging from 10.7% to 74.1%. The analysis revealed three recurring themes contributing to attrition: profession-centric factors (e.g., career progression, job satisfaction, support, and professional growth), systemic-centric factors (e.g., compensation, staffing challenges, clinical practices, patient care, workload), and individual-centric factors (e.g., recognition, the need for change, and burnout).

**Conclusion:**

Attrition in allied health remains a significant challenge. Addressing this issue requires a systemic, nuanced, and evidence-based approach, given the complex, interlinked, and multifaceted factors contributing to attrition. The younger workforce, characterized by changing generational values, necessitates innovative thinking, intersectoral collaboration, and the potential for co-created solutions with, for, and by the allied health workforce.

## Background

On average, human life expectancy has increased by ten years within the last five decades [1]. This respective increase in life expectancy is associated with greater burdens on the healthcare system to manage individuals with non-communicable diseases [2, 3]. According to Philip [1], efficient utilisation of allied health workforce could potentially reduce health system cost burdens by reducing the demand and utilisation of acute health facilities. Additionally, Philip’s research describes the fundamental role of the allied health workforce in chronic disease and multimorbidity management which aligns with current healthcare needs owing to the aging demographics [1]. As morbidity rates continue to change with medical care advancements that facilitate increased life expectancies, a greater employment of allied health professionals is imperative to meet this expanding demand.

According to the United Nations High-Level Commission on Health Employment and Economic Growth, a deficit of 18 million health workers is estimated by 2030 [4]. In addition, the ongoing COVID-19 pandemic has increased the prevalence of psychological distress amongst healthcare workers; contributing to global shortages of health care workers [5]. Furthermore, the health workforce in most developed countries is highly dependent upon foreign health workers. Within Europe, foreign healthcare workers constitute 27% of doctors and 16% of nurses [5]. During the COVID-19 pandemic, the travel restrictions hindered the migration of foreign healthcare workers [5]. A study conducted by Satiani et al. [6] on attrition trends of surgical faculty within a 15-year period in the United States indicated a national turnover between six percent to 12% annually; with 40% of surgeons reporting burnout with intention to leave their current practice [6]. The attrition rates were notably higher amongst women, ethnic minorities, and academic physician professors [6]. Correspondingly, a review conducted by Lopes et al. [7] of 51 academic studies on attrition rates of healthcare workers identified relatively low attrition rates in midwives (4.5% - 16%) and doctors (1.7% - 15%) compared to nurses (4.9% to 44.3%) [7]. Previous studies on attrition amongst allied health professionals indicated higher attrition rates in contrast to other health professions such as general practitioners, dentists, and nurses [8, 9].

At present, there is no globally recognised definition or classification for allied health professionals which presents a challenge for research in this field. In Australia, allied health professionals are described as university qualified practitioners that are not part of medical, dental, or nursing professions [10]. Commonly known allied health professionals include nutritionists, occupational therapists, pharmacists, physiotherapists, psychologists, social workers, podiatrists, audiologists, speech pathologists, and medical radiation professionals [10]. The respective lack of standardisation complicates efforts to address issues such as increased attrition rates among allied health professionals. Previous research indicates that 10% to 15% of Canadian rehabilitation professionals, predominantly occupational therapists and physiotherapists, leave their profession within two years of graduation, and in Australia, 65% of surveyed physiotherapy graduates foresee leaving their profession within the next decade [11]. Contributing factors include heavy caseloads, stress and burnout, desire for increased salary and promotional opportunities, and discrepancies between clinicians’ expectations and actual practice [12]. Understanding these factors is crucial for developing strategies to retain these professionals, thereby ensuring the sustainability of healthcare services. While research highlights important issues with regards to attrition in allied health professions, there is largely a preliminary focus on single disciplines instead of the broader, allied health collective [13–15].

To our knowledge no reviews have been conducted to investigate attrition among all professions within the allied health sector. Furthermore, research on the health workforce has been predominantly focused on physicians and nurses [7, 16]. Therefore, this review aims to map attrition and attrition intention rates, and its attributing factors for allied health professions worldwide [17].

## Method

This scoping review was conducted in accordance with PCC (Population, Concept, Context) [18] framework, which informed its search strategy as recommended by the JBI methodology for scoping reviews [19]. The review followed best practice standards in the conduct and reporting of scoping reviews (PRISMA scoping review) [17]. The protocol of this scoping review has been registered on the Open Science Framework database (DOI 10.17605/OSF.IO/57T3R).

### Search strategy

Preliminary search on MEDLINE and PsycINFO was performed to explore the body of literature and establish key terms and medical subject headings (MeSH) in the field of interest. Search terms, developed based on the population, concept, and context are presented in Table 1. To ensure a rigorous search strategy was developed, the search strategy was evaluated by an academic librarian at the University of South Australia.

**Table 1:** Key concepts and search terms.

Framework Aspects	Search Terms
**Population**	(Allied health or nutrition or dietetic* or “occupational therapy” or physiotherapy or “physical therapy” or psychology or podiatry or social work or pharmacy or medical radiation or audiology or exercise physiology or speech pathology) OR Nutritionists/ or Occupational Therapists/ or Pharmacists/ or Physical Therapists/ or Psychotherapists/ or social workers/ or Podiatry/ or Audiologist/ or Speech Pathologist/ or Exercise physiologist/
**Concept**	(Attrition or “drop out” or drop-out or dropout or burnout or burn-out or “burn out” or “intention to leave” or retention) OR burnout/ or turnover time/
**Context**	(Workforce or “work force” or occupation* or “career mobility”)

A comprehensive search was conducted across seven academic databases (PsycINFO, MEDLINE, Embase, Emcare, CINAHL, Scopus, and the Cochrane Library) from inception to December 2022, supplemented by an updated search on March 18, 2024, to capture current literature. The databases were selected based upon accessibility and applicability to the research question. Search syntax for each database is provided in Appendix A (Tables A1-A7) in S1 Appendix. To reduce the risk of publication bias, Google and Google Scholar were searched for grey literature and the first 10 pages of the search results generated were reviewed [20]. Additionally, organisational websites (Australian Health Practitioner Regulation Agency (APHRA)) and websites of professional societies/associations (in the United States, Europe, United Kingdom, and Australia) were searched for relevant publications. Searches were limited to publications in English. No limitations for age, gender, or country of workplace were applied.

### Eligibility criteria

The following eligibility criteria were developed based upon the predetermined research question (Table 2). Eligibility criteria included studies investigating attrition rates, attrition intention rates, and attrition factors for allied health professionals. Studies involving multiple professions that measured and reported the attrition components on individual allied health professions were included. Studies exploring attrition factors including retirement, disability and/or leaving a workplace but remaining in the profession were excluded.

**Table 2:** Summary of inclusion and exclusion criteria.

	Population	Concept	Context	Studies
**Inclusion**	Allied health professionals (nutritionists, dietitian, occupational therapists, pharmacists, physiotherapist, psychologist, social workers, podiatrist audiologist, speech pathologist, medical radiation professionals, exercise physiologist)	Studies exploring the attribution factors and/or reason for workforce attrition and/or occupational attrition intention. Studies exploring attrition and attrition intention rates within these 10 main allied health workforce	Work settings (hospital, community, residential care etc)	Human English Qualitative/ quantitative studies Published and unpublished studies Grey literature (such as survey results)
**Exclusion**	Non-allied health professionals Students Allied health professional assistant Medical practitioners, dentists, and nurses	Studies exploring changes of jobs within a similar field of occupation or discipline Studies exploring work dissatisfaction and/or excluding attrition Attrition in relation to retirement, death, disability, and/or illness Leaving job/relocating (NOT leaving the profession)	Personal and non-work setting (such as retirement)	Animal Non-English Secondary evidence studies Opinion articles

### Study selection process

Following the search process, results obtained for the databases were uploaded to Endnote 20^TM^ software. Covidence ^TM^ was used to identify duplicate articles and for the screening and selection processes. To select relevant studies, a two-step process was implemented; reviewing the title and abstract followed by screening the full text. Both screening processes were conducted by two independent reviewers (SY and KL/AP/SK) with any screening discrepancies discussed and conflicting votes resolved by a third reviewer.

### Data extraction

A customised data extraction form was developed based on the PCC [18] framework including author, country, health profession, design, method, sample characteristics, attrition rate, intention to leave rates, and factors contributing to attrition (Appendix B, Table B1 in S1 Appendix). Data were extracted independently by SY and reviewed by KL or AP. Discrepancies in data extraction between reviewers were resolved through further review and discussion.

### Data synthesis

Given the nature of the review question, a narrative synthesis of the literature was conducted. To consolidate the quantitative data, summary tables were used, categorising information by profession, attrition rates, intention to leave, and other relevant factors contributing to the attrition of allied health workers from their related professions. The outcomes of each category were thereafter consolidated by contrasting each individual study with their respective allied health professions.

The qualitative data were analysed thematically. Through extensive reading, key themes were compiled from each study and grouped by common traits, allowing central themes to be identified. These themes were collaboratively discussed and refined by the review team until a consensus was achieved. For this review, data from quantitative and qualitative research were broadly categorised into three major themes: i) profession centric (factors related to the profession); ii) system centric (factor related to the overall health system); iii) person centric (factors relating to self/individual). Additional details are provided in the individual sections.

## Results

**Fig 1:**
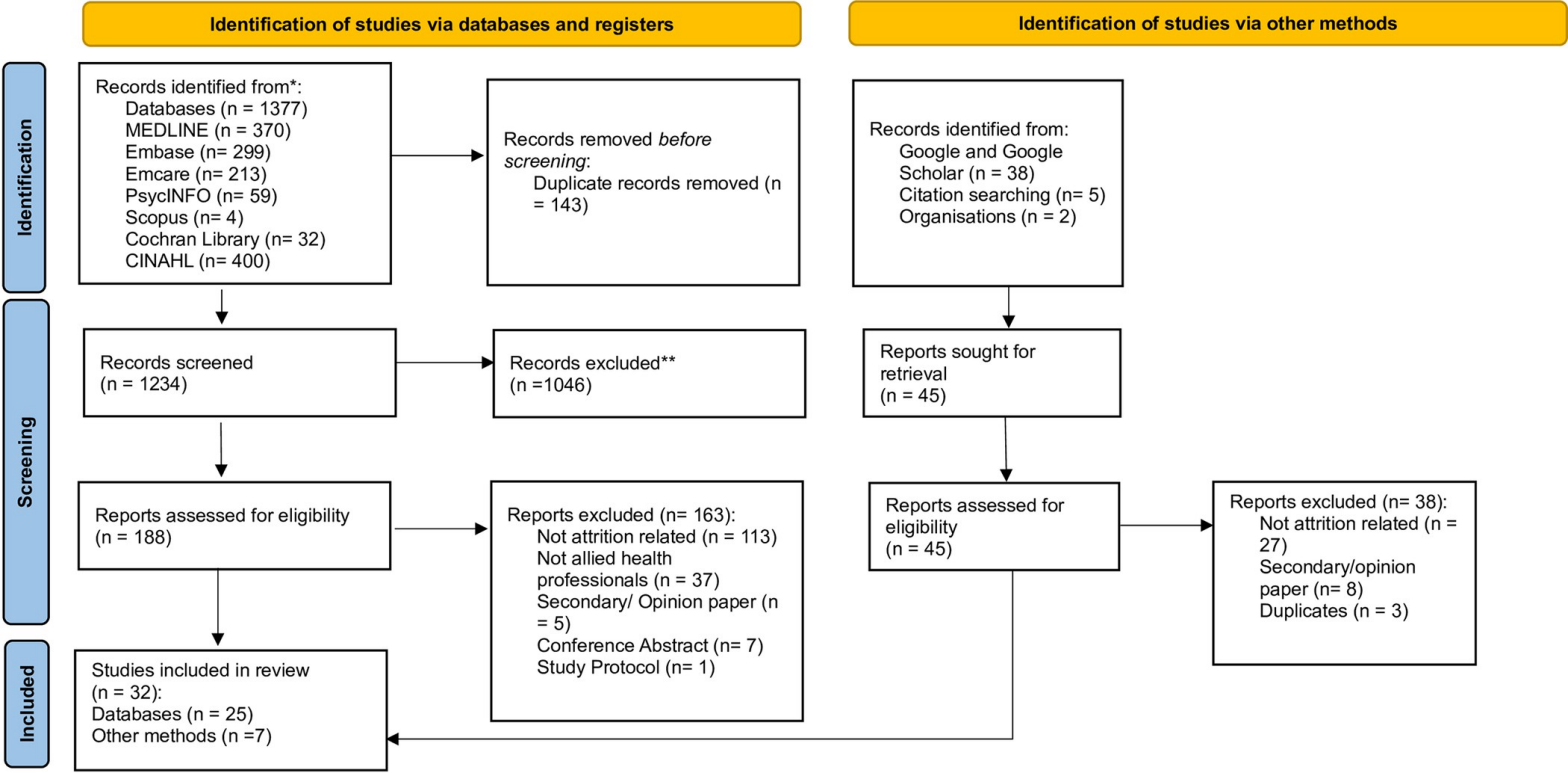
PRSMA flow chart.

The initial search identified 1028 studies, with an additional 394 studies identified from updated searches. Following the removal of duplicates, 1234 studies were screened based on titles and abstracts, resulting in the exclusion of 1046 studies. Of the 233 eligible for full-text screening, 32 studies were included in this scoping review. The results of database searching screening phases, eligibility and rationale for exclusion are presented in Fig 1.

### Characteristics of included studies

The 32 studies included in the scoping review were published between 1990 [21] and 2024 [22] (Table 3). Eleven of the 30 studies were conducted in Australia [8, 14, 23–31], six in the United Kingdom [15, 32–36], five in Canada [13, 37–40], four in the United States of America [21, 41–43], four in New Zealand [22, 44–46], and one in Ireland [47] and Romania [48] respectively. Study designs varied from cross-sectional to qualitative studies as outlined in Table 3. Attrition data were predominantly obtained through survey questionnaires (n = 23) [13, 14, 21–24, 27, 28, 30, 31, 33–42, 45, 47, 48], semi-structured interviews (n = 9) [8, 15, 25, 26, 29, 32, 33, 44, 46], and relevant allied health department data (n = 1) [43]. The professions studied included physiotherapists (n = 7) [24, 28, 29, 31, 38, 40, 44], occupational therapists (n = 6) [13, 21, 27, 33, 37, 47], pharmacists (n = 6) [22, 26, 32, 34, 36, 42], nuclear medicine technologists (n = 5) [15, 23, 25, 35, 41, 45], social workers (n = 2) [39, 48], speech pathologists (n = 2) [8, 14], audiologists (n = 1) [43], podiatrists (n = 2) [30, 46] and multiple allied health professionals (n = 1) [25]. Sample sizes ranged from 12 participants [32] to 32,181 participants [34]. Further characteristics of the studies are outlined in Table 3.

**Table 3:** Study characteristics.

Author, year, and origin	Design	Method	Discipline	Sample size (n)/ Response rate (RR)	Participant characteristics
**Adams et al., 2008, [23] Australia**	Longitudinal observational study	Survey within New South Wales, Australian Capital Territory and Queensland. Data from Census of Population and Housing for 1996 and 2001 and the Australian and New Zealand Society of Nuclear Medicine (ANZSNM).	Nuclear medicine technologist	n = 48 RR = 54%	Age: NR Gender: NR Marital status: NR Qualification: NR Job location: NR Job setting: NR
**Anderson et al., 2005, [24] Australia**	Longitudinal observational study	Survey and data from New South Wales Physiotherapist Registration Board annual report 1987–2002, survey by Department of Labour and Immigration 1975, Census of Population and Housing (1986, 1991, and 1996), and Australian Institute of Health and Welfare (1995 and 2000).	Physiotherapist	1990 (n = 273), 1994 (n = 441), 1998 (n = 532), 1999 (n = 669), 2000 (n = 700), 2001 (n = 718) RR = 80.5%	Age: NR Gender: NR Marital status: NR Qualification: NR Job location: NR Job setting: Own practice 1987 (n = 627, 28.4%), 2001 (n = 1014, 29.6%) Private: 1989 (n = 496, 21.4%), 2001 (n = 742, 24.8%) Public: 1989 (n = 1157, 49.9%), 2001 (n = 1231, 41.2%)
**Bailey, 1990, [21] USA**	Cross-sectional observational study	Postal survey questionnaire	Occupational Therapists	n = 696, RR = 60%	Age: NR Gender: all female Marital status: Single (n = 97, 14%), Married (n = 557, 80%), Divorced/Separated/Widowed (n = 42, 6%) Qualification: NR Job location: NR Job setting: NR
**Beeler et al., 2022, [46] New Zealand**	Descriptive cross-sectional	Video conference/ telephone semi-structured interview	Podiatrist	n = 15	Age: < 30 (n = 2, 13.3%), 30–55 (n = 7, 46.7%), > 55 (n = 6, 40.0%) Gender: Male (n = 4, 26.7%), Female (n = 11, 73.3%) Marital status: NR Qualification: NR Job location: Rural (n = 13, 86.7%), urban (n = 2, 13.3%) Job setting: NR
**Bradley et al., 2024, [22] New Zealand**	Mixed methods cross-sectional	Online survey	Pharmacist	n = 416 RR = 29%	Age: Range = 18–65, mostly in 23–25 (n = 140, 34%) and 26–30 (n = 181, 44%) Gender: Male (n = 101, 24%), Female (n = 304, 73%), Not provided (n = 11, 3%) Marital status: NR Qualification: NR Job location: NR Job setting: Hospital (n = 104, 26%), Community (n = 322, 79%), Distract health board (n = 9, 2%), Primary health organisation (n = 3, <1%), General practice (n = 3, <1%), Academia (n = 12, 3%), Pharmaceutical industry (n = 2, <1%), Other (n = 24, 6%), Not working as pharmacist (n = 7, 2%)
**Brown, 1995, [37] Canada**	Cross-sectional observational study	Postal survey questionnaire	Occupational Therapists	n = 165 RR = 83%	Age: 20–29 (n = 65, 39.3%), 30–39 (n = 69, 42.3%), 40–49 (n = 19, 11.7%), 50–59 (n = 10, 6.1%), 60–69 (n = 1, 0.6%) Gender: all male Marital Status: Single (n = 40, 24.4%), Married (n = 118, 72%), Divorced (n = 2, 1.2%), Separated (n = 1, 0.6%) Qualification: Diploma/certificate (n = 36, 22%), Bachelor’s degree (n = 124, 75.6%), Master’s degree (n = 3, 1.8%, Doctorate (n = 1, 0.6%) Job location: NR Job setting: Hospital (n = 102, 62.2%), Private (n = 4, 2.4%), Public (n = 50, 30.5%), Others (n = 8, 4.9%).
**Couch et al., 2023, [30] Australia**	Cross-sectional analytical	Online survey	Podiatrist	n = 1129 RR = 21%	Age: Mean = 39 Gender: Female (n = 758, 69%) Marital status: NR Qualification: NR Job location: Rural (n = 338, 30%), Metro (n = 791, 70%) Job setting: Private practice (n = 724, 65%), Public health service (n = 394, 35%)
**Eden et al., 2010, [32] UK**	Qualitative study	Semi-structured telephone interviews	Pharmacist	n = 12	Age: Range = 24–32 years Gender: Male 33.3%, Female 66.6% Marital status: NR Qualification: NR Job location: NR Job setting: Hospital (n = 4, 33.3%), Public (n = 5, 41.7%).
**Forbes et al., 2023, [29] Australia**	Descriptive cross-sectional	Semi-structured interview	Physiotherapist	n = 14	Age: Range = 23–42, Mean = 27 Gender: Male (n = 4, 28.6%), Female (n = 10, 71.4%) Marital status: NR Qualification: Job location: NR Job setting: Private (n = 8, 57.1%), Public (n = 4, 28.6%), Hospital (n = 2, 14.3%)
**Jenkins, 1991, [47] Ireland**	Cross-sectional descriptive	Postal survey questionnaire	Occupational Therapists	n = 25	Age: Range = 26–35 years Marital Status: Married 76% Marital status: NR Qualification: NR Job location: NR Job setting: Public (n = 10, 40%)
**Keane et al., 2012, [25] Australia**	Qualitative—grounded theory approach	Semi-structured Interviews	Dietician, optometrist, occupational therapist, Physiotherapist, psychologist, radiographer, social worker, speech pathologist	n = 30	Age: Range = 24–63, Median = 44 years Gender: Female (n = 24, 80%) Marital status: NR Qualification: NR Job location: NR Job setting: Private (n = 5, 17%)
**Laminman, 2007, [13] Canada**	Mix method approach	Self-administered questionnaire	Occupational Therapists	n = 278, RR = 70.7%	Age: Mean = 38.9 years Gender: Female (n = 263, 94.6%) Marital Status: Married (n = 222, 80%) Qualification: NR Job location: City (n = 207, 74.4%), Regional (n = 17, 6%) Job Setting: Hospital (n = 108, 39%), Public (n = 54, 19.6%).
**Lazar et al., 2021, [48] Romania**	Cross sectional	Online survey	Social Workers	n = 1057	Age: <25(n = 64, 6.1%), 26–34 (n = 330, 31.2%), 35–44 (n = 481, 45.5%), 45–54 (n = 156, 14.8%), 55–64 (n = 25, 2.4%), >65 (n = 1, 0.1%) Gender: Male (n = 144, 13.6%), Female (n = 913, 86.4%) Marital Status: Single (n = 244, 23.1%), Relationship (n = 58, 5.5%), Married (n = 689, 65.2%), Divorced (n = 56, 5.3%), Widowed (n = 10, 0.9%). Qualification: Bachelor (n = 1010, 95.6%), Masters (n = 509, 48.2%), Doctorate (n = 53, 5%). Job Location: Urban (n = 937, 88.6%), Rural (n = 120, 11.4%). Job setting: NR
**Mak et al., 2013, [26] Australia**	Qualitative	Semi-structured telephone interview	Pharmacist	n = 20	Age: 20–30 (n = 3, 15%), 31–40 (n = 6, 30%), 41–60 (n = 8, 40%), >60 (n = 3, 15%) Gender: Male (n = 13 65%), Female (n = 7, 35%) Marital status: NR Qualification: NR Job location: NR Job setting: NR
**McLaughlin et al., [8] 2009, Australia**	Qualitative semi-structured interviews	Semi-structured telephone interview	Speech pathologist	n = 18, RR = 30%	Age: NR Gender: all female Marital status: NR Qualification: NR Job location: Rural (n = 7, 38.9%), Metropolitan (n = 11, 61.1%) Job setting: Private (n = 5, 27.8%), Public (n = 13, 72.2%).
**McLaughlin et al., [14] 2010, Australia**	Cross sectional quantitative	Questionnaire	Speech pathologist	n = 620, RR = 21%	Age: 18–25 (n = 60, 13.87%), 26–35 (n = 225, 35.32%), 36–45 (n = 149, 22.74%), 46–55 (n = 106, 16.13%), >56 (n = 22, 2.58%). Gender: Male (n = 14, 2.2%), Female (n = 598, 96.4%). Marital status: NR Qualification: NR Job Location: Metropolitan (n = 451, 72.7%), Non-metropolitan (n = 174, 27.3%) Job setting: Private (n = 201, 32.4%), Public (n = 384, 61.9%)
**Meade et al., 2005, [27] Australia**	Cross sectional descriptive and inferential	Postal survey questionnaire	Occupational Therapists	n = 113, RR = 83%	Age: Range = 20–29 years Gender: Male (n = 46, 40.7%), Female (n = 67, 59.3%) Marital Status: Single (n = 50, 44.2%), Married (n = 60, 53.1%), Divorced (n = 2, 1.8%), Separated (n = 1, 0.9%) Qualification: Diploma/Certificate (n = 3, 2.7%), Bachelor (n = 106, 93.8%), Masters (n = 4, 3.5%) Job location: NR Job setting: Hospital (n = 35, 31%), Private (n = 9, 8%), Public (n = 21, 18.6%).
**Nightingale et al., 2023, [15] UK**	Qualitative pragmatic framework methodology	Semi structured telephone interview	Radiographer	n = 44	Age: NR Gender: NR Marital status: NR Qualification: NR Job location: NR Job setting: NR
**Noh and Beggs, 1993, [38] Canada**	Longitudinal observation	Postal survey questionnaire	Physiotherapist	n = 196, RR = 82%	Age: 22–29 (n = 88, 44.8%), 30–39 (n = 62, 31.6%), >40 (n = 18, 9.1%). Gender: Male (n = 86, 43.8%), Female (n = 55, 28.2%). Marital status: Married (n = 55, 28.3%), Unmarried (n = 63, 32%). Qualification: Diploma (n = 42, 21.5%), Bachelor (n = 68, 34.8%) Job location: NR Job setting: Hospital (n = 74, 37.5%), Private (n = 49, 25%), Public (n = 18, 9.4%).
**Paynter et al., 2023, [31] Australia**	Longitudinal cohort, observational and analytical	Online survey	Physiotherapist	n = 94 RR = 54%	Age: range 23–38, Median: 24.7 Gender: Male (n = 25, 26%) Female (n = 71, 73.9%) Marital status: NR Qualification: NR Job location: NR Job setting: NR
**Rambur et al., 2008, [41] USA**	Cross sectional observational	Two-phase postal survey	Radiographer	n = 241, RR = 51%	Age: Mean = 43.8 years Gender: NR Marital status: NR Qualification: NR Job location: NR Job setting: NR
**Reid and Dixon, 2018, [44] New Zealand**	Mixed method cross-sectional	Semi structured interviews	Physiotherapist	n = 84	Age: NR Gender: NR Marital status: NR Qualification: NR Job location: NR Job setting: NR
**Rugg, 1999, [33] UK**	Cross sectional observation, analytical and qualitative semi-structured interview	Postal questionnaire and semi structured interviews	Occupational Therapists	n = 206	Age: 20–24 (n = 115, 56%), 25–29 (n = 30, 15%), 30–34 (n = 22, 11%), 35–55 (n = 39, 19%) Gender: Female (n = 182, 88%) Marital Status: Unmarried (n = 186, 90%) Qualification: NR Job location: NR Job setting: NR
**Seston et al., 2009, [34] UK**	Longitudinal cohort, observational and analytical	Postal questionnaire	Pharmacist	n = 32181, RR = 76.6%	Age: 21–29 (n = 4882, 22.3%), 30–39 (n = 6173, 28.2%), 40–49 (n = 6061, 27.7%), >50 (n = 4773, 21.8%) Gender: Female (n = 19212, 59.7%) Marital status: NR Qualification: NR Job location: NR Job setting: Hospital (n = 5320, 24.3%), Private (n = 1450, 6.6%), Public (n = 15,119, 69.1%).
**Shier et al., 2012, [39] Canada**	Cross sectional, analytical	Postal survey	Social Workers	n = 145	Age: 19–25 (n = 6, 4.1%), 26–35 (n = 41, 28.3%) 36–45 (n = 38, 26.2%), 46–55 (n = 46, 31.7%), 56–65 (n = 13, 9.0%), >65 (n = 1, 0.7%) Gender: Male (n = 20, 13.8%), Female (n = 125, 86.2%) Marital status: NR Qualification: Diploma (n = 6, 4.1%), Bachelors (n = 111, 76.6%), Masters (n = 21, 14.5%) Job Location: Rural (n = 12, 7%), Small Urban (n = 50, 35%), Large Urban (n = 83, 58%) Job setting: NR
**Skelton et al., 2022, [35] UK**	Cross sectional analytical observation	Online survey questionnaire	Sonographers	n = 138	Age: 21–30 (n = 12, 13.5%), 31–40 (n = 20, 22.5%), 41–50 (n = 24, 27%), 51–60 (n = 31, 34.9%), >61 (n = 2, 2.3%) Gender: Male (n = 2, 2.3%), Female (n = 86, 96.6%), Others (n = 1, 1.1%) Marital status: NR Qualifications: Bachelor (n = 3, 3%), Diploma (n = 5, 5%), Postgraduate (n = 79, 87%), Others (n = 3, 3%) Job location: NR Job setting: NR
**State of Victoria, 2016, [28] Australia**	Analytical cross-sectional observation	Online survey questionnaire	Physiotherapist	n = 1037, RR = 15%	Age: Range = 23–72 years, Mean = 39 years, Median = 37 years Gender: Female (n = 770, 81%) Marital status: NR Qualification: NR Job location: Metropolitan (n = 757, 73%), Job setting: Private (n = 150, 16%)
**Taylor and Oetzel, 2020, [45] New Zealand**	Analytical cross-sectional observation	Online survey	Radiation therapist	n = 362, RR = 91%	Age: <30 (n = 127, 35%), >30 (n = 237, 65%). Gender: Male (n = 47,13%), Female (n = 315, 87%). Marital status: NR Qualification: Certificate (n = 4, 1%), Diploma (n = 65, 19%), Bachelor (n = 233, 77%), Masters (n = 3, 1%), Others (n = 8, 2%) Job location: NR Job setting: Private (n = 57, 17%), Public (n = 267, 78%), Others (n = 18, 5%).
**Wickware, 2022, [36] UK**	Descriptive and cross-sectional observational study	Survey	Pharmacist	n = 291	Age: Range = 55–64 years Gender: NR Marital status: NR Qualification: NR Job location: NR Job setting: NR
**Wilson, 1995, [42] USA**	Descriptive study	Survey	Pharmacy Directors	n = 150	Age: NR Gender: NR Marital status: NR Qualification: NR Job location: NR Job setting: NR
**Windmill and Freemant, [43] 2013, USA**	Predictive or projection	Data from the U.S. Department of Health and Human Services (2006) Physician Supply Model	Audiologist	n = 16000	Age: <30 (n = 1760, 11%), 31–40 (n = 4160, 26%), 41–50 (4000, 25%), 51–60 (n = 4160, 26%), >60 (n = 1920, 12%) Gender: NR Marital status: NR Qualification: NR Job location: NR Job setting: Hospital (n = 1440, 9%), Private (n = 4160, 26%), Education (n = 1920, 12%), Military (n = 480, 3%) Others (n = 8000, 50%)
**Wolpert and Yoshida, 1992, [40] Canada**	Analytical observational study	Postal questionnaire	Physiotherapist	n = 601 Cancelled, n = 165 RR 74%, Inactive, n = 217 76%, Control, n = 219 RR = 77%	Age: Range = 24–82, Mean: 42 Gender: Male (n = 28, 7.3%) Marital status: Married (n = 303, 79%), Separated/Divorced (n = 21, 5.5%), Single (n = 58, 15.1%) Qualification: Diploma (n = 172, 44.9%), Bachelor (n = 211, 55.1%), Master (n = 25, 6.5%) Job location: NR Job setting: NR

### Quantitative results

#### Attrition rates

Twenty-five studies presented quantitative findings, detailed in Table 4. Among them, nine studies provided attrition rates, ranging from 0.5% [34] to 41% [43]. The lowest attrition rates were observed among pharmacists between 0.5% [34] and 1% [42]. For physiotherapists, attrition rates varied, ranging from 1.6% [38] to 12% [24]. The highest attrition rate, at 41%, was reported in a study involving audiologists [43]. A study provided attrition rates for nuclear medicine technologists from 1996–2001, broken down by age group, with the 45–49 years category having the highest attrition rate within the profession [23]. However, attrition rates for social workers, medical radiation professionals, and speech pathologists were not reported.

**Table 4:** Quantitative attrition results.

Author	Health Profession	Attrition Rate	Attrition Intention Rate
Bailey [21]	Occupational Therapists	NR	NR
Brown [37]	Occupational Therapists	NR	74.1%
Jenkins [47]	Occupational Therapists	NR	32%
Laminman [13]	Occupational Therapists	NR	10.7%
Meade, Brown [27]	Occupational Therapists	NR	60%
Rugg [33]	Occupational Therapists	5%	24%
Anderson, Ellis [24]	Physiotherapist	4.8% (2001), 5% (1991)	NR
Noh and Beggs [38]	Physiotherapist	1.6%	NR
Paynter et al. [31]	Physiotherapist	NR	15%
Reid and Dixon [44]	Physiotherapist	12%	NR
State of Victoria [28]	Physiotherapist	NR	27%
Wolpert and Yoshida [40]	Physiotherapist	NR	NR
Seston, Hassell [34]	Pharmacist	0.5%	8.7%
Bradley et al. [22]	Pharmacist	NR	44%
Wilson [42]	Pharmacy Directors	1%	NR
Lazar, Lightfoot [48]	Social Worker	NR	20.5%
Shier, Graham [39]	Social Worker	NR	NR
Beeler et al. [46]	Podiatrist	6.7%	NR
Couch et al. [30]	Podiatrist	NR	21%
Adams, Schofield [23]	Nuclear medicine technologist	12%	NR
McLaughlin, Adamson [14]	Speech pathologist	NR	NR
Rambur, Palumbo [41]	Radiographer	NR	7.6%
Skelton, Harrison [35]	Sonographers	NR	12.5%
Taylor and Oetzel [45]	Radiation therapist	NR	35%
Windmill and Freeman [43]	Audiologist	41%	NR

#### Intention to leave

The intention to leave the profession was more frequently reported than actual attrition rates, as shown in Table 4. Among the 14 studies [13, 22, 27, 28, 30, 31, 33–35, 37, 41, 45, 47, 48] that examined participants’ intent to leave their profession, a study involving radiographers reported the lowest intention to leave at 7.6% [41]. Conversely, studies observing occupational therapists reported the highest intent to leave, with rates ranging from 10.7% [13] to 74.1% [37]. Three professions, including nuclear medicine technologists, audiologists, and speech pathologists, did not report any intention to leave the profession.

### Factors contributing to attrition

Ten studies examined factors contributing to attrition, as summarised in Table 5. The most commonly cited reasons for attrition included job dissatisfaction (n = 7) [13, 21, 30, 34, 39, 40, 45], lack of autonomy (n = 4) [13, 21, 34, 40], burnout (n = 2) [40, 48], and overburdened workload (n = 4) [13, 21, 34, 45]. Studies also indicated that male pharmacists [34] and nuclear medicine [23] technologists were more inclined to leave their professions compared to their female counterparts. Furthermore, social workers who exhibited higher levels of occupational commitment reported greater job satisfaction and expressed less intent to leave their profession [39]. Age played a role in attrition, with varying findings across three studies [21, 34, 45]. Specifically, Bailey [21] noted that younger age groups with less than three years of professional occupational therapy experience had attrition rates of 54%. Conversely, older radiation therapists reported higher job satisfaction and lower intent to leave the profession [45]. Additionally, pharmacists in their 20s and 50s were more likely to consider leaving their profession compared to those in their 30s [34].

**Table 5:** Factors contributing to attrition.

Study	Person centric						Profession centric					System centric					
	Personal	Family	Need for change	Recognition	Conflict of values	Burnout	Career Pathway	Job Satisfaction	Autonomy	Support	Professional Development	Staffing	Patient Care	Clinical Practice	Bureaucracy	Workload	Remuneration
Adams, Schofield [23]	✓																
Bailey [21]	✓	✓	✓	✓	✓		✓	✓	✓	✓			✓		✓	✓	✓
Couch et al. [30]								✓		✓							
Laminman [13]				✓				✓	✓						✓	✓	
Lazar, Lightfoot [48]						✓											✓
McLaughlin, Adamson [14]		✓															
Seston, Hassell [34]								✓	✓							✓	
Shier, Graham [39]								✓									
Taylor and Oetzel [45]	✓		✓					✓								✓	
Wolpert and Yoshida [40]		✓	✓	✓		✓		✓	✓		✓						✓

### Qualitative results

**Fig 2:**
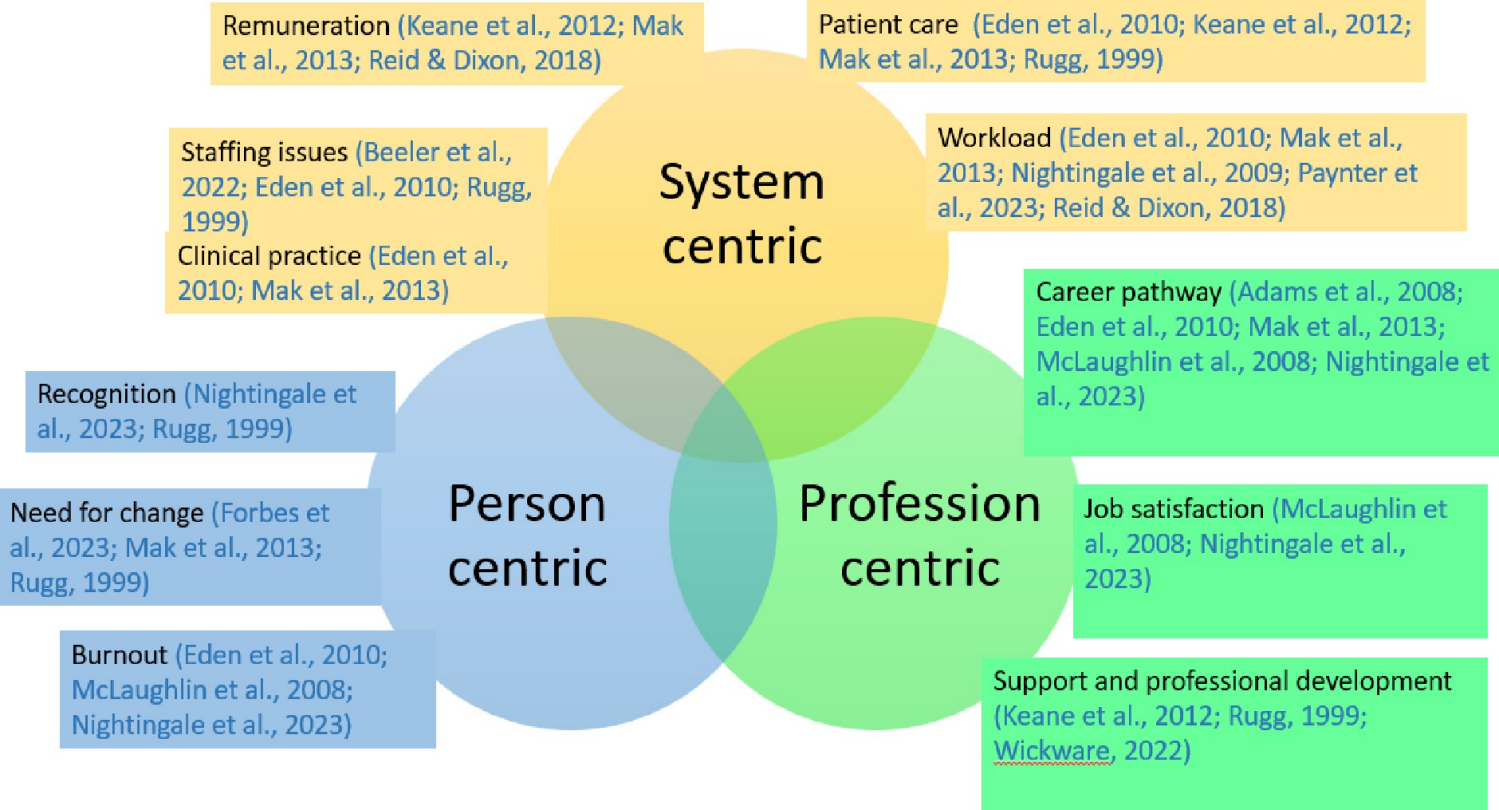
Major themes from qualitative data.

Qualitative data were reported by 12 of the 31 included studies [8, 15, 23, 25, 26, 29, 31–33, 36, 44, 46]. Thematic analysis of the studies revealed three major overarching themes: i) profession centric; ii) system centric; iii) person centric (Fig 2). As discussed, the first central theme, profession centric was consolidated from factors related to shortcomings identified by participants in relation to the profession involving subthemes of: i) lack of career pathway; ii) job dissatisfaction; and iii) lack of support and professional development. The second theme, system centric, involved factors related to the overall healthcare system with subthemes of: i) impact of workload; ii) barriers to optimal patient care; iii) staffing issues; iv) limited clinical practice; and v) remuneration. The third theme, person centric encompasses personal factors involving subthemes of: i) lack of recognition; ii) need for change; and iii) burnout. Nine of the qualitative studies reported profession-centric themes [8, 15, 23, 25, 26, 32, 33, 36, 44] while eight reported system centric themes [15, 25, 26, 31–33, 44, 46] and six reported personal centric themes [8, 15, 26, 29, 32, 33].

#### Theme 1: Profession centric

*Lack of career pathway*. The most frequently described profession centric factor was the lack of career pathway, and the related insufficient career opportunities within clinical practices [8, 15, 23, 26, 32]. The absence of a satisfactory career pathway prompted frustration and reduced career motivations within allied health professionals. This factor was observed in five studies expressed by nuclear medicine technologist [23], pharmacists [26, 32], speech pathologists [8], and radiographers [15]. Lack of career progression was associated with feelings of *“frustration”* [32] ^(p.185)^, *“overwhelm”* [26] ^(p.133)^, and being *“blocked”* [15] ^(p.78)^.

Changing professions was denoted as a result of the lack of career progression and opportunity to professionally progress. Several health professionals discussed their dissatisfaction with their available career pathways:

*‘‘I was at the top of the grading, and so there was really no where for me to go except to move out of the profession…”* (Speech pathologist) [8] ^(p.167)^

*“Lack of career pathways in nuclear medicine*. *No succession planning*. *No chief jobs*. *Reach senior then a dead end so technologist looks for another career”*. (Nuclear medicine technologist) [23] ^(p.289)^

*Job satisfaction*. Job satisfaction factors describe the extent of contentment and fulfilment experienced by professionals in performing their job responsibilities. Two studies involving speech pathologists [8] and radiographers [15] highlighted job dissatisfaction as a key factor contributing to attrition. Speech pathologist expressed a sense of being unable to *“make a difference”* [8] ^(p.166)^, leading to a desire to pursue a different profession where they could feel more *“valued”* [8] ^(p.166)^ and have a greater *“impact”* [8] ^(p.166)^. Meanwhile, radiographers expressed discontent with the repetitive nature of their job:

*“You were just kind of factory workers, and that wasn’t the buzz for the job, and I struggled doing the same thing, and working on the same machine day in day out… It wasn’t for me.”* (Radiographer) [15] ^(p.78)^

*Lack of support and professional development*. Lack of support and professional development factors revolves around deficiency in resources, guidance, or mentorship, professional development training, and job performance reviews. Three studies involving occupational therapists [33], pharmacists [36], and physiotherapists [25] identified issues related to the absence of support and professional development factors. Occupational therapists characterised the lack of support as *"dreadful"*, *"devalued*,*"* and *"neglected*,*"* [33] ^(p.289)^. Additionally, occupational therapists associated the absence of opportunities for personal development with a sense of incompetency:

*“When I asked if there were any courses, I was told ‘no way’. I didn’t seem to be able to get any further… I wouldn’t feel competent to go on to a Senior post, I wouldn’t feel that I’d learnt any more than a Basic Grade.”* (Occupational therapist) [33] ^(p.290)^

Physiotherapists and pharmacists involved in the studies cited *"no support"* [25] ^(p.8)^ and a *"lack of protected time"* [36] ^(p.2)^ for professional development as reasons for their decision to leave their respective professions.

#### Theme 2: System centric factors

*Impact of workload*. Impact of workload entails the significant influence that the quantity and nature of work responsibilities have on individuals’ decisions to leave their professions. This theme encompasses components such as excessive work demands, overwhelming job pressures, and an imbalance between workload and available resources. Workload was a major system centric factor contributing to attrition and intent to leave amongst pharmacists [26, 32], radiographers [15], and physiotherapists [31, 44]. A radiographer highlighted a system that *“does not accept the fact that you are getting older”* [15] ^(p.80)^ and is not designed to accommodate an aging workforce in terms of workload. Pharmacists expressed concerns of carrying out their responsibilities *“effectively”* [32] ^(p.184)^ and *“safely”* [32] ^(p.184)^ within limited time constraints. Workloads were further described by pharmacists and physiotherapists as:

*“You know, long hours, no breaks, pays pretty ordinary and the level of responsibility and stress with the amount of prescriptions that you’re expected to do…it’s just like working in a factory… I felt like a glorified factory worker…”* (Pharmacist) [26] ^(p.133)^

*“Most new graduates said they weren’t prepared for the heavy workloads or the psychosocial aspect of face-to-face client interaction*.*”* (Physiotherapist) [44] ^(p.23)^

*“Long work hours and giving up my own sport and hobbies to pursue this career*.*”*(Physiotherapist) [31] ^(p.10)^

*Barriers to optimal patient care*. Barriers to optimal patient care involve challenges and limitations of professionals when striving to provide optimal patient care; that is ethically and professionally acceptable. This theme emerges as the second major system centric factor expressed in four studies by physiotherapists [25], pharmacists [26, 32], and occupational therapists [33]. Physiotherapists voiced concerns about the expectation of patient care, deeming it "professionally compromising" [25] ^(p.7)^ and "disparate to the needs of the client," [25] ^(p.7)^ leading to attrition from the profession. Pharmacists also expressed feelings of dissatisfaction regarding similar issues, highlighting the pervasion of patient care constraints [26, 32]. Occupational therapists similarly conveyed challenges related to optimal patient care:

*“I’m actually leaving the rotation to find a post somewhere else… I find it incredibly frustrating that you don’t get to follow… [patients]… through.”* (Occupational therapist) [33] ^(p.289)^

*Impact of staffing*. The effects and consequences of staffing-related factors on the decision of professionals to leave their respective professions is prevalent. This respective theme explores how staffing issues, such as insufficient personnel, high workload, or inadequate support, contribute to challenges and difficulties faced by professionals in delivering quality care. Both pharmacists [32] and occupational therapists [33] voiced apprehensions that revolved around the challenges posed by staff shortages:

*“Yeah. I mean they want to maximise profits, the large companies, because the shareholders want maximum profits… maximum output and minimum staff.”* (Pharmacist) [32] ^(p.184)^

*“*… *got me to take an unqualified member of staff*, *and teach them the ropes*, *and then put them in my place*… *without any supervision or any help*, *having to give information to doctors and nurses*… *She just filled a gap*,*”* (Occupational therapist) [33] ^(p.290)^

Podiatrists additionally described that shortages in personnel resulted in insufficient time off:

“*There are not enough podiatrists … Not even close”* [46] ^(p.6)^

“*I never really went away… any time I had off I worked around Christmas or long weekends*” [46] ^(p.6)^

*Limited clinical practice*. Limited clinical practice refers to the impact of constrained or restricted opportunities for professionals to engage in hands-on, practical, and clinically relevant duties within their field. This theme explores how a lack of exposure to diverse and meaningful clinical practices can contribute to professionals’ dissatisfaction, reduced skill development, overall frustration and eventual decision to leave their profession. Pharmacists expressed their lack of opportunity to utilise their clinical skills [26] and characterised their role as primarily focused on *“dispensing and checking”* [pharmaceuticals] [32] ^(p.184)^. Pharmacists also expressed dissatisfaction with the misalignment between the perceived role during training:

*“We’re taught in University which I think is wrong, that we have a certain clinical role…but in community [pharmacy], that role doesn’t exist.”* (Pharmacist) [26] ^(p.134)^

*Insufficient remuneration*. Insufficient remuneration refers to the impact of low or insufficient salaries and benefits on dissatisfaction and frustration among individuals in the profession. It encompasses the economic aspects of the job, including salary structures, benefits packages, and overall compensation, and examines how these factors may influence professionals’ job satisfaction, motivation, and ultimately their decision to pursue alternative career paths. Frustration stemming from inadequate remuneration or wages, despite possessing skill competency in their respective professions, was identified in three studies involving pharmacists [26], physiotherapists [44], and speech pathologists [25]. Insufficient remuneration devalued the roles of pharmacists, as expressed:

*“…when you go for a position, there’s not really much opportunities to negotiate, because what’s basically put on you is like, if you don’t want this job, some other script monkey will do it for a lot less.”* (Pharmacist) [26] ^(p.133)^

#### Theme 3: Person centric factor

*Lack of recognition*. Lack of recognition refers to the perceived insufficiency of acknowledgment and appreciation of the contributions, skills, and efforts of professionals within their respective fields. This theme explores how professionals may feel undervalued or overlooked in terms of their expertise, achievements, and the overall impact of their work. This lack of recognition can result in decreased job satisfaction, demotivation, and contribute to the decision to leave the profession; in search of environments where their contributions are appreciated and acknowledged. Lack of recognition was expressed in two studies involving occupational therapists [33] and radiographers [15]. Occupational therapists conveyed instances of being excluded from discussions and the expectation to entertain patients, which resulted in feelings of incompetence [33]. In the meantime, radiographers highlighted the adverse impact of a lack of recognition on their decision to discontinue practice:

*“And I think that if that contribution was at least acknowledged by anyone I would have felt better about things, I would have definitely felt better about staying… no appreciation for all the hard work…”* (Radiographer) [15] ^(p.78)^

*Need for change*. The need for change comprises professionals’ dissatisfaction with the overall structure, policies, and practices of their employment; contributing to a lack of fulfillment and prompting profession changes. This respective theme emerged in three studies, encompassing pharmacists [26], occupational therapists [33] and physiotherapist [29]. The intentions behind the decision to change professions was described by healthcare professionals:

*“I’m looking forward to moving on because… there’s not enough challenge in… [this job]… I would like to be in a job where I get to use all… [my]… skills, that’s a bit more challenging…”* (Occupational therapist) [33] ^(p.290)^

*“I did have a second pathway that I was alternatively going to take*… *it was almost a flip of a coin there*. *And that other pathway has continued to nudge at me as well*. *So there’s that option to take that and it’s sort of a bit of a fear of missing out*.*”* (Physiotherapist) [29] ^(p. 6)^

*Burnout*. The theme of burnout refers to a state of chronic physical and emotional exhaustion, often accompanied by feelings of cynicism and detachment from work. Professionals experiencing burnout find themselves overwhelmed by prolonged and intense workplace stress, resulting in a reduced sense of personal accomplishment, diminished interest in their professional roles, and ultimately the desire to leave their profession. Radiographers [15], pharmacists [32], and speech pathologists [8] in three studies highlighted the risk of burnout. Pharmacists denoted *“dreading going into work”* as a direct result of an overwhelming workload [32] ^(p.184)^. A speech pathologist identified that *“if the negatives outweigh your positives*, *then I think you’ll find people are really dissatisfied and getting burned out and leaving the profession*…*”* (Speech pathologist), denoting the primary consequences of burnout for many healthcare professionals [8] ^(p.166)^.

## Discussion

The aim of this scoping review was to map the literature on attrition rates and contributory factors of attrition within global allied health professions. Within 32 studies identified within this scoping review, attrition and attrition intention rates were examined in research with audiologists [43], nuclear medicine technologists [23], radiographers [41], radiation therapists [45], physiotherapists [24, 28, 29, 31, 38, 44], occupational therapists [13, 27, 33, 37, 47], pharmacists [22, 34, 42], podiatrists [30, 46], social workers [48], and sonographers [35]. Occupational therapists and physiotherapists were the central focus of attrition research among allied health professionals. Accordingly, attrition studies involving nutritionists, and psychologists were not identified within this review. The rate at which allied health professionals withdrew from their professions varied considerably (0.5% [34] to 74.1% [37]) amongst professions. Thus, indicating potential factors that contribute to attrition across allied health disciplines. These factors were broadly categorised into profession-centric [13, 21, 34, 39, 40, 45], system-centric [13, 21, 34, 45, 48], and person-centric [13, 14, 21, 23, 40, 45, 48] factors.

The influential interconnected nature of attrition factors within allied health professionals were a chief finding derived from this review [15]. Factors such as the lack of recognition and need for change, were expressed simultaneously, indicating an interconnected relationship between person centric factors [33]. Furthermore, the need for change was described concurrently with the absence of career pathways within profession centric factors [26]. Similarly, system centric factors, particularly the impact of workload, were concurrently expressed alongside profession-centric factors like job satisfaction and person-centric factors such as burnout. Conclusively, attrition and attrition intention amongst allied health disciplines are not solely influenced by individual factors but rather, a multitude of factors distributed across various "levels," with each factor and level influencing the others. These findings are shared by similar research amongst other health disciplines. A systematic review of 19 studies exploring attrition factors among physicians highlighted the potential to introduce a multifaceted approach to mitigate attrition; providing financial incentives, career development, sufficient staffing, maintenance of professional work environments, manageable workloads, and autonomy, with recommendations for a multifaceted approach to tackle this challenge [49].

Attrition amongst allied health professionals remains a persistent global challenge. According to McLaughlin, Lincoln [8], 52% of speech pathologists in Queensland, Australia, expect to stay in the profession for less than 10 years. Similarly, Pretorius, Karunaratne [9] reported that 60% of physiotherapy graduates in Australia anticipate leaving the workforce within 10 years. While evidence-informed strategies to address attrition should be implemented, these findings also raise the question of whether career changes among allied health professionals signify a potential "new normal" in the future of the healthcare workforce, largely a result of changes in generational personal and work values. A study examining shifts in career values across generations identified significant differences between millennials and their predecessors [50]. Notably, millennials placed greater importance on intellectual stimulation, followed by advancements, workplace social interactions, job prestige, and the importance of having fun, with a crucial need for a work-life balance, distinctive from the values of earlier generations [50]. Additionally, younger generations considered work as less central in their lives, prioritised leisure more, and exhibited different work ethics compared to older counterparts [51].

These findings have important consequences for the future of allied health workforce. First, the allied health workforce should recognise, cater to and support those who seek career changes and non-linear career paths. This could be achieved by ensuring allied health professionals are inclusive of diverse positions, have structured and supported career pathways and, include opportunities for progression outside of frontline clinical practice (such as leadership roles, joint appointments with educational institutions) [52]. Policies that establish and promote organisational values, especially by those in leadership roles, can further enhance job satisfaction and retention. Additionally, formal support programs such as mentorships in the first year of practice have shown significant value in retaining professionals by providing essential guidance and support [29, 31]. Second, the values of self-care and work-life balance embraced by the younger generation may directly conflict with the demanding and emotionally taxing nature of the role as a health professional (such as the increasing prevalence of burnout) [50]. As a result, the conventional expectation for healthcare professionals to uphold a "lifelong" commitment to the health workforce may no longer be relevant for the younger generation of healthcare professionals. While this issue may not be completely resolvable, the younger generation’s work life may be extended by ensuring professional support and mentoring through clinical supervision, peer support and career pathways in complementary fields (such as leadership and management) [53]. Furthermore, targeted initiatives such as rural admissions schemes for training programs can help address workforce shortages and health inequalities in underserved areas [46].

The transformation, and evolving career values of, the health workforce has been expedited by the COVID-19 pandemic [54]. Research by Skelton, Harrison [35] reported that 12.5% of sonographers’ intended to leave their profession during the COVID-19 outbreak, driven by job dissatisfaction, burnout, and psychological distress. These findings are supported by previous research including Bhardwaj [55], which reflected a 10% increase in burnout amongst physicians in 2021, with 50% of physicians reporting burnout amidst the pandemic. Lou, Montreuil [56] reported an attrition intention rate of 20% among physicians in Canada during the pandemic in contrast to 3.2% amongst physicians in the United Kingdom prior to COVID-19 [57]. Similarly, burnout was identified as the major theme contributing to attrition amongst registered nurses in Italy and Greece during COVID-19 [58, 59]. In line with this, 49.3% of nurses in Ghana intend to leave the profession because of burnout amidst the COVID-19 pandemic [60]. Collectively, these studies underscore the transformative impact of the COVID-19 pandemic on the global health workforce and advocate for a thoughtful and nuanced approach to health workforce planning [61].

### Strengths and limitations

This review adhered to best practice guidelines throughout the conduct and reporting of scoping reviews (PRISMA-ScR). Despite efforts to minimise publication bias within grey literature and citation searches, the review only included studies published in English, potentially introducing publication and language bias in study selection, as relevant studies in languages other than English were excluded. While grey literature was searched via organisational and professional society websites, these were mostly confined to Western jurisdictions (e.g., United States, Europe, United Kingdom, and Australia). Therefore, this introduces a significant limitation of the review as publications and other resources from developing countries may have been overlooked. Data on certain disciplines were not identified in this review and therefore the collective extent of attrition concerning the allied health workforce could not be captured. All studies identified in this review were undertaken in developed countries and two thirds were published over a decade ago (prior to the impact of the COVID-19 pandemic), and thus, the generalisability to the global allied health workforce within the current health care context is limited. Future research should explore attrition, intention to leave, and contributing factors among nutritionists, and psychologists to mitigate the information deficit regarding these professions and increase breadth of research. Additionally, investigating attrition amongst allied health professionals in the post-COVID-19 world, will enhance understandings of the pandemic’s impact on this essential workforce.

## Conclusion

The ongoing concerns about the allied health workforce and its capacity to meet increasing health care demands has fuelled interest and research on this topic. Despite the findings of this research reflecting variable attrition rates across health disciplines, the contributing factors are consistently complex and interlinked; thus, requiring a systemic, nuanced and evidence-informed approach.

The literature additionally posits the potential existence of a “new-normal” for the allied health workforce, involving the changes in generational values amongst younger workers; driven by the COVID-19 pandemic. These respective changes comprise a younger workforce pursuing flexibility in work conditions, fun, opportunities for career progression, self-care interests and work-life balance. Ultimately, satisfying these demands will require innovative thinking, intersectoral collaboration and introduce the potential of co-created solutions with, for, and by the allied health workforce.

## Supporting information

S1 Appendix (DOCX)
