# Supplementary material for: Unveiling the Exodus: A scoping review of attrition in allied health
Source: PLoS One. 2024 Sep 6;19(9):e0308302. doi: 10.1371/journal.pone.0308302 (PMC11379274; doi:10.1371/journal.pone.0308302)
Supplement: S1 Appendix — (DOCX) [file pone.0308302.s001.docx]

**Appendices**

**Appendix A**

**Table A1.** Search Syntax for Ovid MEDLINE

| # | Search | Results from 11/12/2022 |
| --- | --- | --- |
| 1 | Allied Health Personnel/ | 12881 |
| 2 | Allied Health Occupations/ | 609 |
| 3 | ("allied health" or "allied health personnel*" or "allied health occupation*").tw. | 11334 |
| 4 | 1 or 2 or 3 | 23092 |
| 5 | Nutritionists/ | 1697 |
| 6 | (dieti* or nutrition*).tw. | 339178 |
| 7 | 5 or 6 | 339498 |
| 8 | Occupational Therapy/ | 14619 |
| 9 | "occupational therap*".tw. | 16119 |
| 10 | 8 or 9 | 22400 |
| 11 | Exercise Therapy/ | 47966 |
| 12 | ("exercise therap*" or "exercise physiolog*" or physiotherap* or "physical therap*").tw. | 62373 |
| 13 | 11 or 12 | 103110 |
| 14 | psycholog*.tw. | 323880 |
| 15 | psychotherap*.tw. | 47321 |
| 16 | 14 or 15 | 362575 |
| 17 | podiatr*.tw. | 3243 |
| 18 | Radiography/ or Radiology/ | 348534 |
| 19 | (radiographer* or radiologist*).tw. | 62106 |
| 20 | 18 or 19 | 401330 |
| 21 | radiotherapy/ | 44224 |
| 22 | "radiation therap*".tw. | 87221 |
| 23 | 21 or 22 | 123422 |
| 24 | Pharmacology/ | 33483 |
| 25 | pharmac*.tw. | 872249 |
| 26 | 24 or 25 | 899728 |
| 27 | "social work*".tw. | 17712 |
| 28 | audiolog*.tw. | 11984 |
| 29 | Speech-Language Pathology/ | 3617 |
| 30 | "speech patholog*".tw. | 1538 |
| 31 | 28 or 29 or 30 | 16489 |
| 32 | 4 or 7 or 10 or 13 or 16 or 17 or 20 or 23 or 26 or 27 or 31 | 2243733 |
| 33 | attrition.tw. | 15524 |
| 34 | "attrition intention*".tw. | 5 |
| 35 | "personnel turnover".tw. | 68 |
| 36 | ("drop out" or dropout or "drop-out").tw. | 16523 |
| 37 | turnover.tw. | 105999 |
| 38 | Burnout, Professional/ | 16375 |
| 39 | "intention to leave".tw. | 775 |
| 40 | (burnout or "burn out" or "burn-out").tw. | 18863 |
| 41 | 33 or 34 or 35 or 36 or 37 or 38 or 39 or 40 | 160975 |
| 42 | Workforce/ or Health Workforce/ | 80350 |
| 43 | "career mobility".tw. | 147 |
| 44 | 42 or 43 | 80477 |
| 45 | 32 and 41 and 44 | 327 |

**Table A2.** Search Syntax for Ovid Emcare

| # | Search | Results from 11/12/2022 |
| --- | --- | --- |
| 1 | Allied Health Personnel/ | 3544 |
| 2 | Allied Health Occupations/ | 40140 |
| 3 | ("allied health" or "allied health personnel*" or "allied health occupation*").tw. | 8136 |
| 4 | 1 or 2 or 3 | 51056 |
| 5 | Nutritionists/ | 5594 |
| 6 | (dieti* or nutrition*).tw. | 152871 |
| 7 | 5 or 6 | 153575 |
| 8 | Occupational Therapy/ | 12654 |
| 9 | "occupational therap*".tw. | 15653 |
| 10 | 8 or 9 | 19417 |
| 11 | Exercise Therapy/ | 6825 |
| 12 | ("exercise therap*" or "exercise physiolog*" or physiotherap* or "physical therap*").tw. | 42974 |
| 13 | 11 or 12 | 48006 |
| 14 | psycholog*.tw. | 208694 |
| 15 | psychotherap*.tw. | 23483 |
| 16 | 14 or 15 | 226420 |
| 17 | podiatr*.tw. | 2079 |
| 18 | Radiography/ or Radiology/ | 32681 |
| 19 | (radiographer* or radiologist*).tw. | 41248 |
| 20 | 18 or 19 | 70311 |
| 21 | radiotherapy/ | 23268 |
| 22 | "radiation therap*".tw. | 29557 |
| 23 | 21 or 22 | 44934 |
| 24 | Pharmacology/ | 3876 |
| 25 | pharmac*.tw. | 214018 |
| 26 | 24 or 25 | 214851 |
| 27 | "social work*".tw. | 22246 |
| 28 | audiolog*.tw. | 6653 |
| 29 | Speech-Language Pathology/ | 494 |
| 30 | "speech patholog*".tw. | 1307 |
| 31 | 28 or 29 or 30 | 8380 |
| 32 | 4 or 7 or 10 or 13 or 16 or 17 or 20 or 23 or 26 or 27 or 31 | 816199 |
| 33 | attrition.tw. | 7587 |
| 34 | "attrition intention*".tw. | 3 |
| 35 | "personnel turnover".tw. | 34 |
| 36 | ("drop out" or dropout or "drop-out").tw. | 8125 |
| 37 | turnover.tw. | 17945 |
| 38 | Burnout, Professional/ | 552 |
| 39 | "intention to leave".tw. | 707 |
| 40 | (burnout or "burn out" or "burn-out").tw. | 13181 |
| 41 | 33 or 34 or 35 or 36 or 37 or 38 or 39 or 40 | 45861 |
| 42 | Workforce/ or Health Workforce/ | 7400 |
| 43 | "career mobility".tw. | 55 |
| 44 | 42 or 43 | 7453 |
| 45 | 32 and 41 and 44 | 116 |

**Table A3.** Syntax for Ovid Embase

| # | Search | Results from 11/12/2022 |
| --- | --- | --- |
| 1 | Allied Health Personnel/ | 16136 |
| 2 | Allied Health Occupations/ | 61366 |
| 3 | ("allied health" or "allied health personnel*" or "allied health occupation*").tw. | 14882 |
| 4 | 1 or 2 or 3 | 90293 |
| 5 | Nutritionists/ | 16382 |
| 6 | (dieti* or nutrition*).tw. | 468604 |
| 7 | 5 or 6 | 470606 |
| 8 | Occupational Therapy/ | 28402 |
| 9 | "occupational therap*".tw. | 25808 |
| 10 | 8 or 9 | 38012 |
| 11 | Exercise Therapy/ | 36160 |
| 12 | ("exercise therap*" or "exercise physiolog*" or physiotherap* or "physical therap*").tw. | 107009 |
| 13 | 11 or 12 | 135223 |
| 14 | psycholog*.tw. | 470558 |
| 15 | psychotherap*.tw. | 73288 |
| 16 | 14 or 15 | 527949 |
| 17 | podiatr*.tw. | 4224 |
| 18 | Radiography/ or Radiology/ | 346786 |
| 19 | (radiographer* or radiologist*).tw. | 99489 |
| 20 | 18 or 19 | 432687 |
| 21 | radiotherapy/ | 205318 |
| 22 | "radiation therap*".tw. | 144944 |
| 23 | 21 or 22 | 296078 |
| 24 | Pharmacology/ | 66217 |
| 25 | pharmac*.tw. | 1360710 |
| 26 | 24 or 25 | 1405859 |
| 27 | "social work*".tw. | 28793 |
| 28 | audiolog*.tw. | 16280 |
| 29 | Speech-Language Pathology/ | 23764 |
| 30 | "speech patholog*".tw. | 2535 |
| 31 | 28 or 29 or 30 | 41637 |
| 32 | 4 or 7 or 10 or 13 or 16 or 17 or 20 or 23 or 26 or 27 or 31 | 3336303 |
| 33 | attrition.tw. | 20485 |
| 34 | "attrition intention*".tw. | 6 |
| 35 | "personnel turnover".tw. | 89 |
| 36 | ("drop out" or dropout or "drop-out").tw. | 24386 |
| 37 | turnover.tw. | 132800 |
| 38 | Burnout, Professional/ | 2488 |
| 39 | "intention to leave".tw. | 777 |
| 40 | (burnout or "burn out" or "burn-out").tw. | 23957 |
| 41 | 33 or 34 or 35 or 36 or 37 or 38 or 39 or 40 | 200490 |
| 42 | Workforce/ or Health Workforce/ | 13726 |
| 43 | "career mobility".tw. | 158 |
| 44 | 42 or 43 | 13876 |
| 45 | 32 and 41 and 44 | 163 |

**Table A4.** Search Syntax from Ovid PsycINFO

| # | Search | Results from 11/12/2022 |
| --- | --- | --- |
| 1 | Allied Health Personnel/ | 1290 |
| 2 | Allied Health Occupations/ | 0 |
| 3 | ("allied health" or "allied health personnel*" or "allied health occupation*").tw. | 3149 |
| 4 | 1 or 2 or 3 | 4026 |
| 5 | Nutritionists/ | 0 |
| 6 | (dieti* or nutrition*).tw. | 34351 |
| 7 | 5 or 6 | 34351 |
| 8 | Occupational Therapy/ | 7028 |
| 9 | "occupational therap*".tw. | 12310 |
| 10 | 8 or 9 | 13069 |
| 11 | Exercise Therapy/ | 0 |
| 12 | ("exercise therap*" or "exercise physiolog*" or physiotherap* or "physical therap*").tw. | 8947 |
| 13 | 11 or 12 | 8947 |
| 14 | psycholog*.tw. | 659040 |
| 15 | psychotherap*.tw. | 131265 |
| 16 | 14 or 15 | 755192 |
| 17 | podiatr*.tw. | 135 |
| 18 | Radiography/ or Radiology/ | 1091 |
| 19 | (radiographer* or radiologist*).tw. | 645 |
| 20 | 18 or 19 | 1579 |
| 21 | radiotherapy/ | 0 |
| 22 | "radiation therap*".tw. | 1168 |
| 23 | 21 or 22 | 1168 |
| 24 | Pharmacology/ | 10882 |
| 25 | pharmac*.tw. | 96433 |
| 26 | 24 or 25 | 98079 |
| 27 | "social work*".tw. | 49666 |
| 28 | audiolog*.tw. | 3098 |
| 29 | Speech-Language Pathology/ | 1442 |
| 30 | "speech patholog*".tw. | 1261 |
| 31 | 28 or 29 or 30 | 5495 |
| 32 | 4 or 7 or 10 or 13 or 16 or 17 or 20 or 23 or 26 or 27 or 31 | 932774 |
| 33 | attrition.tw. | 9552 |
| 34 | "attrition intention*".tw. | 14 |
| 35 | "personnel turnover".tw. | 92 |
| 36 | ("drop out" or dropout or "drop-out").tw. | 12349 |
| 37 | turnover.tw. | 14893 |
| 38 | Burnout, Professional/ | 0 |
| 39 | "intention to leave".tw. | 842 |
| 40 | (burnout or "burn out" or "burn-out").tw. | 16600 |
| 41 | 33 or 34 or 35 or 36 or 37 or 38 or 39 or 40 | 51290 |
| 42 | Workforce/ or Health Workforce/ | 3338 |
| 43 | "career mobility".tw. | 285 |
| 44 | 42 or 43 | 3619 |
| 45 | 32 and 41 and 44 | 30 |

**Table A5.** Search Syntax for CINAHL

| # | Search | Results from 11/12/2022 |
| --- | --- | --- |
| S1 | (allied health or nutrition or dietetic* or "occupational therapy" or physiotherapy or "physical therapy" or psychology or podiatry or social work or pharmacy or medical radiation or audiology or exercise physiology or speech pathology)  Search mode: AB Abstract | 58242 |
| S2 | (attrition or "drop out" or drop-out or dropout or burnout or burn-out or "burn out" or "intention to leave" or retention or “career mobility”)  Search mode: AB Abstract | 51310 |
| S3 | (workforce or "work force" or occupation*)  Search mode: AB Abstract | 84982 |
| S4 | S1 and S2 and S3 | 315 |

**Table A6.** Syntax for The Cochran Library

| # | Search | Results from 11/12/2022 |
| --- | --- | --- |
| S1 | (allied health or nutrition or dietetic* or "occupational therapy" or physiotherapy or "physical therapy" or psychology or podiatry or social work or pharmacy or medical radiation or audiology or exercise physiology or speech pathology)  Search mode: Tittle Abstract Keyword | 2077 |
| S2 | (attrition or "drop out" or drop-out or dropout or burnout or burn-out or "burn out" or "intention to leave" or retention)  Search mode: Tittle Abstract Keyword | 1155 |
| S3 | (workforce or "work force" or occupation* or “career mobility”)  Search mode: Tittle Abstract Keyword | 175 |
| S4 | S1 and S2 and S3 | 30 |

**Table A7.** Syntax for Scopus

| # | Search | Results from 11/12/2022 |
| --- | --- | --- |
| S1 | (allied health or nutrition or dietetic* or "occupational therapy" or physiotherapy or "physical therapy" or psychology or podiatry or social work or pharmacy or medical radiation or audiology or exercise physiology or speech pathology)  Search mode: Article Tittle, Abstract, Keyword | 22 |
| S2 | (attrition or "drop out" or drop-out or dropout or burnout or burn-out or "burn out" or "intention to leave" or retention)  Search mode: Article Tittle, Abstract, Keyword | 639196 |
| S3 | (workforce or "work force" or occupation* or “career mobility”)  Search mode: Article Tittle, Abstract, Keyword | 713334 |
| S4 | S1 and S2 and S3 | 4 |

**Search Syntax for Google Scholar and Google**

Key words include: “allied health professional”, “attrition”, and “attributing factors”.

**Appendix B**

| Data Extractor | Data Checked By | Study No (Covidence Number) | Author | Year | Name of Study | Country | Design | Method of attrition related data collection (e.g. survey, focus group, census data) | Allied Health job title | Ethics approval | Response rate if survey | Participant demographic details? | Qualitative results - factors resulting in attrition | Quantitative results - attrition rate |
| --- | --- | --- | --- | --- | --- | --- | --- | --- | --- | --- | --- | --- | --- | --- |
|  |  |  |  |  |  |  |  |  |  |  |  |  |  |  |
|  |  |  |  |  |  |  |  |  |  |  |  |  |  |  |
|  |  |  |  |  |  |  |  |  |  |  |  |  |  |  |
|  |  |  |  |  |  |  |  |  |  |  |  |  |  |  |
|  |  |  |  |  |  |  |  |  |  |  |  |  |  |  |
|  |  |  |  |  |  |  |  |  |  |  |  |  |  |  |

**Table B1.** Data Extraction
